# Transcriptome analysis of ovary tissues from low- and high-yielding Changshun green-shell laying hens

**DOI:** 10.1186/s12864-021-07688-x

**Published:** 2021-05-14

**Authors:** Ren Mu, Yi-yin Yu, Tuya Gegen, Di Wen, Fen Wang, Zhi Chen, Wen-bin Xu

**Affiliations:** 1 College of Biological Science and Agriculture, Qiannan Normal University for Nationalities Duyun, Jianjiang Road 5, 558000 Duyun, China; 2 Library, Qiannan Normal University for Nationalities, 558000 Duyun, China; 3 College of Animal Sciences, Zhejiang University, 310058 Hangzhou, China; 4 School of Marine Sciences, Ningbo University, 315211 Ningbo, China

**Keywords:** Changshun green-shell laying hens, Egg production, Ovary, Transcriptome analysis

## Abstract

**Background:**

Changshun green-shell laying hens are unique to Guizhou Province, China, and have high egg quality. Improving egg production performance has become an important breeding task, and in recent years, the development of high-throughput sequencing technology provides a fast and exact method for genetic selection. Therefore, we aimed to use this technology to analyze the differences between the ovarian mRNA transcriptome of low and high-yield Changshun green-shell layer hens, identify critical pathways and candidate genes involved in controlling the egg production rate, and provide basic data for layer breeding.

**Results:**

The egg production rates of the low egg production group (LP) and the high egg production group (HP) were 68.00 ± 5.56 % and 93.67 ± 7.09 %, with significant differences between the groups (*p* < 0.01). Moreover, the egg weight, shell thickness, strength and layer weight of the LP were significantly greater than those of the HP (*p* < 0.05). More than 41 million clean reads per sample were obtained, and more than 90 % of the clean reads were mapped to the *Gallus gallus* genome. Further analysis identified 142 differentially expressed genes (DEGs), and among them, 55 were upregulated and 87 were downregulated in the ovaries. KEGG pathway enrichment analysis identified 9 significantly enriched pathways, with the neuroactive ligand-receptor interaction pathway being the most enriched. GO enrichment analysis indicated that the GO term transmembrane receptor protein tyrosine kinase activity, and the DEGs identified in this GO term, including PRLR, NRP1, IL15, BANK1, NTRK1, CCK, and HGF may be associated with crucial roles in the regulation of egg production.

**Conclusions:**

The above-mentioned DEGs may be relevant for the molecular breeding of Changshun green-shell laying hens. Moreover, enrichment analysis indicated that the neuroactive ligand-receptor interaction pathway and receptor protein tyrosine kinases may play crucial roles in the regulation of ovarian function and egg production.

**Supplementary Information:**

The online version contains supplementary material available at 10.1186/s12864-021-07688-x.

## Introduction

Chicken eggs are an important food resource for humans as they contain high-quality protein, essential vitamins and minerals, and are inexpensive. Global egg consumption has tripled in the past 40 years and this trend is predicted to continue [1]. The question of how to increase egg production has consequently become a critically important question for the egg industry.

Improving the genetic potential of chickens is one of the most important strategies utilized to increase egg production. However, conventional breeding techniques based on long-term selection that use egg numbers and laying rate are usually laborious and time-consuming [2]. Currently, various high-throughput techniques that can identify genes at the genomic and transcriptomic levels have been increasingly employed for studying poultry reproduction. For instance, in goose ovaries, twenty-six genes were identified that may be related to the egg-laying process by using suppression subtractive hybridization and reverse dot-blot analysis [3]. Using a large-scale transcriptome sequencing technique, five genes in the ovarian tissues of *Anser cygnoides* were identified that may play important roles in determining high reproductive performance [4]. In chicken, nine genes (BDH, NCAM1, PCDHA, PGDS, PLAG1, PRL, SAR1A, SCG2, and STMN2) related to high egg production levels in the hypothalamus and pituitary gland were identified using a cDNA chip [5]. In another study, the hypothalamus and pituitary expression profiles in high- and low-yield laying chickens were analyzed by RNA-seq [6]. Seven and 39 differentially expressed genes (DEGs) were identified in the hypothalamus and pituitary, respectively, and were associated with the amino acid metabolism, glycosaminoglycan biosynthesis, and estrogen negative feedback systems. Using cDNA microarray analysis, TXN, ACADL, ING4, and ANXA2 were reported to express at higher levels in the ovarian follicles of high-yielding chicken [7]. By comparing the transcriptomes in ovarian tissues of chickens that showed greater and lesser egg-producing capacity, five candidate genes were identified to related to egg production, including ZP2, WNT4, AMH, IGF1, and CYP17A1 [8]. In addition, Mishra et al. [2] performed RNA-seq to explore the chicken transcriptome in the hypothalamic-pituitary-ovarian (HPO) axis. Their results showed that 414, 356 and 10 DEGs were identified in the pituitary gland, ovary, and hypothalamus, respectively, between high and low-yielding chickens. These DEGs were involved in the regulation of the mTOR and Jak-STAT signaling pathways, tryptophan metabolism and PI3K-Akt signaling pathways at the HPO axis. High throughput techniques provide a fast and exact method for genetic selection and have the potential to be an appealing alternative to conventional breeding techniques.

Changshun green-shell chickens are native breeds found in Guizhou province, China. They are dual-purpose egg and meat-type chickens, and their eggs have extremely high economic value, owing to their appearance, higher protein content, better amino acid composition, and lower fat content [9]. The Hartz unit of Changshun green shell eggs is 76.39 ± 2.76 (Level AA), which is extremely significantly better than that of white shell eggs (*p* < 0.01), and the calcium and phosphorus content are significantly higher, while the crude fat content is significantly lower than that of white shell eggs (*p* < 0.01) [10]. Our previous study also found similar results (data unpublished). However, as an indigenous chicken breed, Changshun green-shell chicken shows relatively low egg production [11]. Thus, the purpose of this study was to investigate the mechanisms that affect the egg production of Changshun green-shell chickens. Towards this end, we conducted high-throughput RNA sequencing in the ovaries of Changshun green-shell chickens, to (1) determine the differences in the ovary mRNA transcriptomes between the low- and high-yielding Changshun green-shell laying hens, and (2) identify the critical pathways and candidate genes involved in controlling the egg production rate.

## Materials and methods

### Ethics statement

This study was carried out in accordance with the recommendations in the Guide for the Care and Use of Laboratory Animals of the Ministry of Science and Technology of the People’s Republic of China, and followed the Regulations for the Administration of Affairs Concerning Experimental Animals, Qiannan Normal University for Nationalities (Guizhou, China). and in compliance with ARRIVE 2.0 guidelines. The animal protocol was approved by the Animal Ethics Committee of the Qiannan Normal University for Nationalities.

### Animal and sample preparation

A total of 80 Changshun green-shell layers raised in the poultry breeding farm of Qiannan Normal University for Nationalities were used in this study. At the beginning of the study (age of 240 days), the layers had similar body weights of 1.36 ± 0.14 kg. All layers were housed individually in the battery cages (36 cm-width × 48 length × 38 height) with the same feeding and management conditions throughout the study period. The room temperature was maintained at 22 ± 2℃. The light regime was 16 L:8D. Layers were allowed ad libitum access to diet and water. Diet was supplemented three times daily (7:00, 13:00, and 19:00). Egg number and egg weight were recorded every day (16:00). At 290 days of age, four high-yield (high egg production group, HP) and four low-yield individuals (low egg production group, LP) were selected from the batch of laying hens according to their laying rates. The eggshell thickness and strength were evaluated daily. In the early morning at the age of 300 days, the chickens were anesthetized using sodium pentobarbital after weighing, and ovarian samples were obtained after slaughter. All samples were immediately frozen in liquid nitrogen and stored at -80 °C until analysis. The layers had fasted overnight before being sampled.

### RNA extraction, cDNA library construction, and mRNA sequencing

Total RNA from eight individuals in the two different groups (HP and LP) was extracted from the ovary samples using the Trizol reagent (Takara Bio, Dalian, China), according to the manufacturer’s instructions. In total, eight samples were obtained (one sample per individual). The concentration and quality of the total RNA were determined using a NANOdrop ND-2000 spectrophotometer (Thermo Scientific, Wilmington, DE, USA) and electrophoresis. Sample integrity was evaluated using a microfluidic assay on the Bioanalyzer (Agilent Technologies, Inc., Santa Clara, CA, USA). Library construction and RNA sequencing were performed as a fee-for-service by GENEWIZ, Inc. (Suzhou, China). Briefly, mRNAs were enriched using magnetic beads with Oligo (dT) and were randomly fragmented using a fragmentation buffer. The first strand of cDNA was synthesized with a random hexamer-primer using the mRNA fragments as a template. The second strand of cDNA synthesis was then performed using the Buffer, deoxynucleotide triphosphates (dNTPs), ribonuclease H (RNase H), and DNA polymerase I. The cDNA was purified with a QiaQuick PCR extraction kit (Qiagen, Germany) and eluted with elution buffer for end repair and poly (A) addition. Sequencing adapters were ligated to the 5′ and 3′ ends of the fragments. The fragments were purified using agarose gel electrophoresis and enriched by PCR amplification to obtain a cDNA library. The cDNA libraries were loaded on an Illumina sequencing platform (NovaSeq 6000) for sequencing.

### Data analysis

Quality control checks for the raw reads were performed using FastQC (v0.11.5). Raw reads were trimmed using the fastx_trimmer (fastx_toolkit-0.0.13.2) to obtain clean reads. Clean reads were subsequently mapped against the chicken reference genome *Gallus gallus* (v6.0) that was available in Ensembl v98 using HiSAT2 (v2.2.1) with default parameters. Raw counts of the genes were obtained using the htseq-count package (v0.12.3) in Python (v3.5). Raw counts were normalized using the DESeq2 package (v1.28.1) in R (v4.0.2) to obtain the gene expression level. The overall similarity between the samples was assessed using principal component analysis (PCA) in R (v4.0.2).

### Identification of differentially expressed genes

The differentially expressed genes (DEGs) were identified using the DESeq2 package (v1.28.1) in R (v4.0.2). Genes with an adjusted *p*-value ≤ 0.05 and |Log_2_Fold Change| ≥ 1 were assigned as differentially expressed. Hierarchical clustering and heatmaps of the DEGs were obtain using the Pheatmap package (v1.0.12) in R (v4.0.2).

### KEGG pathway and gene ontology (GO) enrichment analysis

KEGG pathway (Kyoto Encyclopedia of Genes and Genomes; http://www.genome.jp/kegg/) [12] and GO (http://geneontology.org) enrichment analysis of the DEGs were performed using the clusterProfiler package (v3.16.1) in R (v4.0.2), with an adjusted *p* < 0.05 as the screening standard.

### Gene expression analysis by qRT-PCR

Using total RNA, one µg was reverse transcribed to cDNA using the Prime Script RT reagent Kit (Takara Bio, Dalian, China). The mRNA expression values of six candidate genes were randomly selected from the DEGs, and analyzed to verify the RNA-sequencing results. β-actin was chosen as an internal control for the normalization of expression levels. The primers used in the qRT-PCR were designed using Primer 5 (Table 1).

**Table 1:** Primers used for qRT-PCR

Gene Symbol	Gene Name	Primer Sequence (5’-3’)	Accession Number
OVA	ovalbumin	F: CACAAGCAATGCCTTTCAGA	NM_205152.2
		R: GACTTCATCAGGCAACAGCA	
OVALX	ovalbumin-related protein X	F: AAGATCCTGGAGCTCCCATT	NM_001276386.1
		R: CTCCATGGTATTGGGATTGG	
OVALY	ovalbumin-related protein Y	F: GCAAACCTGTGCAAATGATG	NM_001031001.1
		R: GTCTTCTCAATCCGCTCCAG	
AMN	amnion associated transmembrane protein	F: GCTCTGGGTTCACAGCTTTC	NM_001277516.1
		R: TGGAAGATGACGTGGTCGTA	
POMC	proopiomelanocortin	F: AAGGCGAGGAGGAAAAGAAG	XM_015285103.2
		R: CTTTTGACGATGGCGTTTTT	
CGA	glycoprotein hormones	F: AGGGTTGTCCAGAGTGCAAG	NM_001278021.1
		R: TCTTGGTGAAAGCCTTTGCT	
β-actin	beta-actin	F: GAGAAATTGTGCGTGACATGA	NM_205518.1
		R: CCTGAACCTCTCATTGCCA	

Gene expression was analyzed using the ABI7900 system (ABI7900 Applied Biosystems, USA), and the AceQ qPCR SYBR Green Master Mix (Vazyme Biotech Co., Ltd, China). The PCR protocol was initiated at 95 °C for 10 min, followed by 40 cycles of the amplification program, with denaturation at 95 °C, 15 s, and annealing/extension at 60 °C, 60 s. At the end of the last amplification cycle, melt curves were generated to confirm the specificity of the amplification reaction. Each assay was carried out in triplicate and included a negative control. Relative quantification of the gene expression was performed using the 2^−ΔΔCt^ method.

### Statistical analysis

Statistical analyses were performed using the R software (v4.0.2, R Development Core Team 2019). Data were analyzed using the Student’s t-test after testing for the homogeneity of variance with Levene’s test. All data are presented as the mean ± SD, and a *p* < 0.05 was considered statistically significant.

## Results

### Body weight, egg production, and egg quality

Details for the body weight, egg production, and egg quality are shown in Table 2. The laying rates were significantly higher in the HP than LP group (93.67 ± 7.09 vs. 68.00 ± 5.56, *p* < 0.01). However, egg weight, shell thickness, and strength were greater (*p* < 0.05) in the LP than in the HP group. In addition, the final body weight was higher (*p* < 0.05) in the LP group than in the HP group.

**Table 2:** Body weight, egg production and egg quality of low- and high-yielding Changshun green-shell laying hen

	Treatment		Sig
	LP	HP	
Initial body weight (g)	1.46 ± 0.08	1.27 ± 0.13	NS
Final body weight (g)	1.54 ± 0.07	1.26 ± 0.15	*
Laying rate (%)	68.00 ± 5.56	93.67 ± 7.09	**
Egg weight (g)	46.91 ± 0.45	45.04 ± 1.02	*
Eggshell thickness (mm)	0.30 ± 0.02	0.29 ± 0.06	*
Shell strength (N/cm^2^)	39.22 ± 0.30	37.97 ± 0.69	*

*, *p* < 0.05; **, *p* < 0.01; *NS* no significant difference, *LP* low egg production group; *HP* high egg production group

### RNA sequencing quality assessment

The quality metrics of the transcriptomes are shown in Table 3. A total of 8 cDNA libraries were constructed from the ovaries of the Changshun green-shell laying hens. The raw reads and clean reads of each library were more than 42 and 41 million, respectively, except for HP-2, which had ~ 39.2 million raw reads and 39.0 million clean reads. The GC content of all samples ranged from 49.11 to 52.24 %, the base percentage of the Q20 was above 97.79 %, and the percentage of the Q30 base was above 93.55 %. In summary, the sequencing data was suitable for subsequent data analysis.

**Table 3:** Quality metrics of transcripts in the ovary of Changshun green-shell laying hen

Samp	Raw reads	Clean reads	Clean bases	Q20 (%)	Q30 (%)	GC (%)	N (ppm)
LP-1	45,772,004	45,672,822	6,793,131,642	97.90	93.84	49.52	4.74
LP-2	45,989,900	45,890,650	6,822,515,847	97.82	93.66	49.61	4.69
LP-3	45,847,818	45,755,138	6,808,443,826	98.15	94.48	49.82	4.77
LP-4	43,101,534	43,023,412	6,400,232,494	98.07	94.20	50.19	5.75
HP-1	47,619,274	47,511,052	7,060,329,437	98.19	94.65	52.24	4.68
HP-2	39,165,330	39,090,800	5,819,349,384	98.13	94.39	49.47	4.72
HP-3	42,022,532	41,943,412	6,246,359,132	97.79	93.55	49.11	5.73
HP-4	44,730,920	44,654,676	6,649,244,662	98.05	94.16	50.24	5.73

*Samp* Sample name; *Q20* sequencing error rates lower than 1 %; *Q30* sequencing error rates lower than 0.1 %; *GC* the percentage of G and C bases in all transcripts; *N* unknown base; *LP* low egg production group; *HP* high egg production group

### Transcriptome alignment

The results of the trimming and read mapping are shown in Table 4. The total mapped ratio between the reads and the reference genome of all the samples ranged from 90.30 to 92.37 %. The uniquely mapped ratio ranged from 86.59 to 88.89 %. The results indicated that the transcriptome data were reliable and suitable for subsequent analysis.

**Table 4:** Summary of trimming and read mapping results

Samp	Total reads	Total mapped	Multiple mapped	Uniquely mapped
LP-1	45,672,822	41,835,034 (91.60 %)	1,582,978 (3.47 %)	40,252,056 (88.13 %)
LP-2	45,890,650	41,787,491 (91.06 %)	1,616,242 (3.52 %)	40,171,249 (87.54 %)
LP-3	45,755,138	41,901,902 (91.58 %)	1,585,544 (3.47 %)	40,316,358 (88.11 %)
LP-4	43,023,412	39,740,455 (92.37 %)	1,496,311 (3.48 %)	38,244,144 (88.89 %)
HP-1	47,511,052	42,941,478 (90.38 %)	1,803,134 (3.80 %)	41,138,344 (86.59 %)
HP-2	39,090,800	35,970,409 (92.02 %)	1,342,878 (3.44 %)	34,627,531 (88.58 %)
HP-3	41,943,412	38,492,966 (91.77 %)	1,432,361 (3.41 %)	37,060,605 (88.36 %)
HP-4	44,654,676	41,115,072 (92.07 %)	1,619,095 (3.63 %)	39,495,977 (88.45 %)

*Samp* Sample name; *LP* low egg production group; *HP* high egg production group

### Differentially expressed genes

**Fig. 1:**
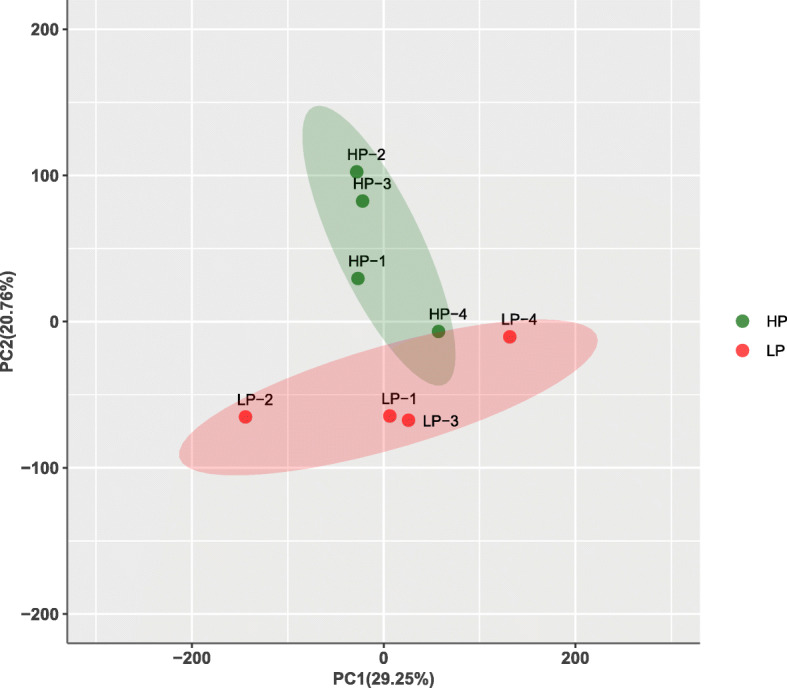
PCA score plot of ovary transcriptomes. HP, high egg production group; LP, low egg production group. Green point, samples from HP; Red point, samples from LP

**Fig. 2:**
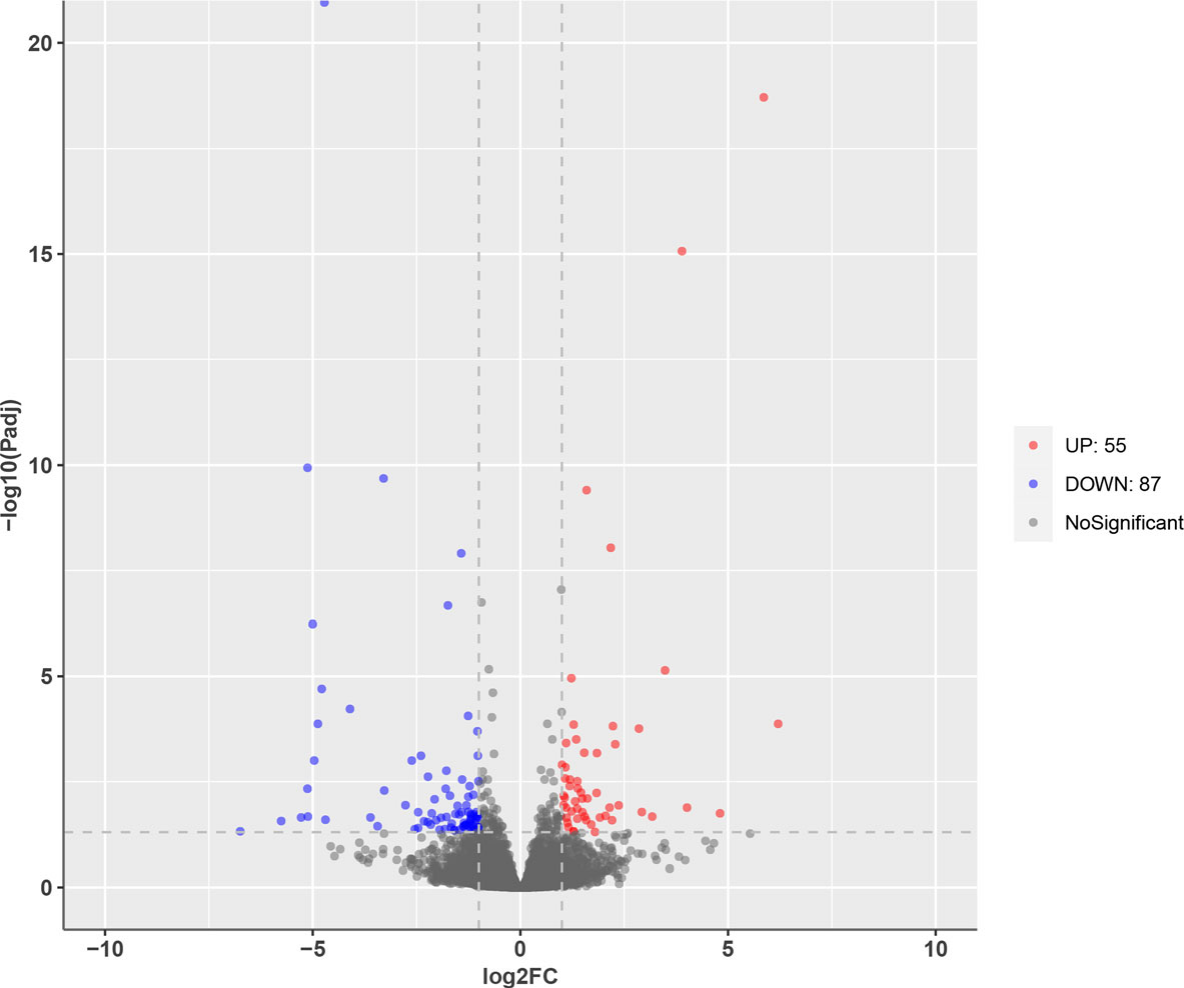
Volcano plot of all expressed genes. The red plots represent significantly upregulated genes; the blue plots represent significantly down − regulated genes; the gray plot represents genes with no significance

**Fig. 3:**
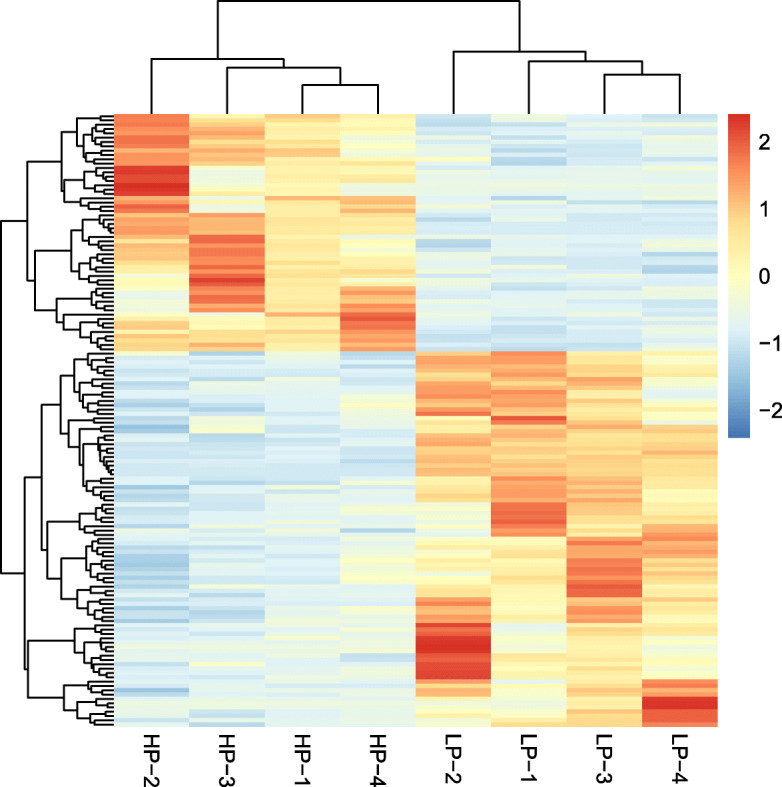
Hierarchical clustering analysis of DEGs. HP, high egg production group; LP, low egg production group

Samples were first analyzed using PCA. In general, the samples from the different groups were divided into two parts in the PCA score plots except for HP-4, which partially overlapped with the LP group (Fig. 1), indicating an obvious difference between the LP and HP groups. A total of 142 DEGs were identified, including 55 upregulated genes and 87 downregulated genes in the HP group (Fig. 2). The DEGs were subsequently analyzed by hierarchical clustering analysis. Samples from the same group were clustered together, and the heatmap visually reflected the differences in the gene expression patterns between the LP and HP groups (Fig. 3).

### KEGG pathway and GO enrichment analysis

**Fig. 4:**
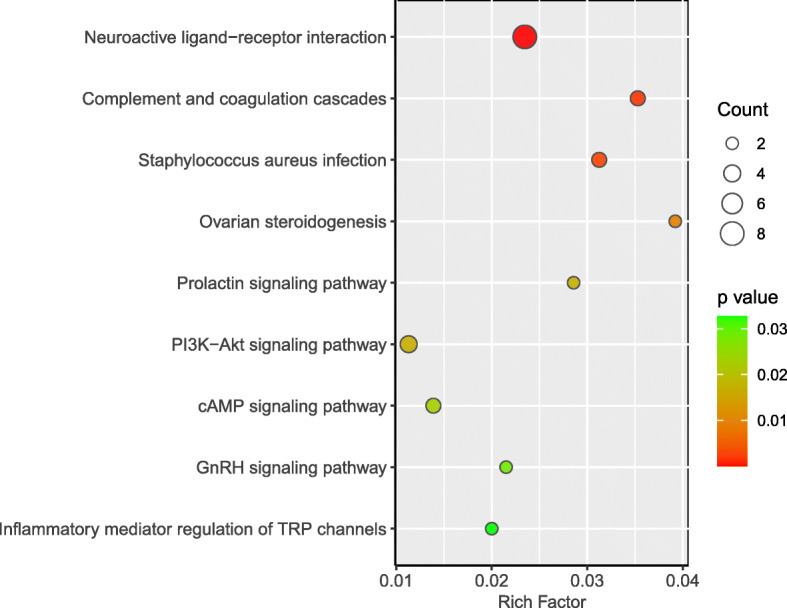
KEGG pathway enrichment analysis of DEGs. Count, number of DEGs enriched in the pathway

**Fig. 5:**
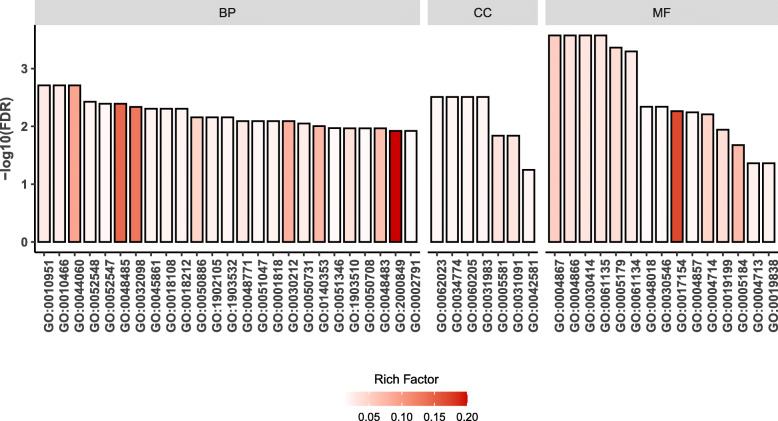
GO enrichment analysis of DEGs. BP, biological processes; CC, cellular components; MF, molecular function

To further elucidate the biochemical functions of the DEGs, we performed KEGG pathway enrichment analysis and GO enrichment analysis. Fifty-three out of 142 DEGs annotated by OrgDb were used for enrichment analysis. A total of nine KEGG pathways were significantly enriched (*p* < 0.05, Fig. 4) including those for neuroactive ligand-receptor interaction, complement and coagulation cascades, *Staphylococcus aureus* infection, ovarian steroidogenesis, prolactin signaling pathway, PI3K − Akt signaling pathway, cAMP signaling pathway, GnRH signaling pathway, and inflammatory mediator regulation of TRP channels. The descriptions of these KEGG pathways are given in Table 5. A total of 220 GO terms were significantly enriched (FDR < 0.05), and most of them belonged to biological processes (BP), followed by molecular functions (MF), and cellular components (CC). The top 25 significantly enriched GO terms for BP as well as all the significantly enriched GO terms for MF and CC are shown in Fig. 5. The descriptions of these GO terms are given in Tables S1, S2, S3. The top 25 significantly enriched BP GO terms were mainly related to the regulation of peptidase activity and endocrine process, regulation of secretion, and lipid export from cell. The significantly enriched CC GO terms were collagen-containing extracellular matrix, secretory granule lumen, cytoplasmic vesicle lumen, vesicle lumen, collagen trimer, platelet alpha granule, and specific granule. The significantly enriched MF GO terms included peptidase regulator and inhibitor activity, receptor ligand activity, transmembrane receptor protein kinase activity, and growth factor binding.

**Table 5:** The significantly enriched KEGG pathway

Description	Rich Factor	P value	Q value	Gene
Neuroactive ligand-receptor interaction	0.0235	2.72E-06	1.49E-04	PRLR/FSHB/C3AR1/F2RL1/ CGA/POMC/GALR1/CCK
Complement and coagulation cascades	0.0353	1.74E-03	4.49E-02	FGG/C3AR1/C1QA
Staphylococcus aureus infection	0.0313	2.46E-03	4.49E-02	FGG/C3AR1/C1QA
Ovarian steroidogenesis	0.0392	9.18E-03	1.26E-01	FSHB/CGA
Prolactin signaling pathway	0.0286	1.68E-02	1.54E-01	PRLR/CGA
PI3K-Akt signaling pathway	0.0113	1.69E-02	1.54E-01	PRLR/MYB/NTRK1/HGF
cAMP signaling pathway	0.0139	2.28E-02	1.79E-01	FSHB/CGA/POMC
GnRH signaling pathway	0.0215	2.87E-02	1.96E-01	FSHB/CGA
Inflammatory mediator regulation of TRP channels	0.0200	3.28E-02	1.99E-01	F2RL1/NTRK1

### qRT-PCR validation of RNA-Seq results

**Fig. 6:**
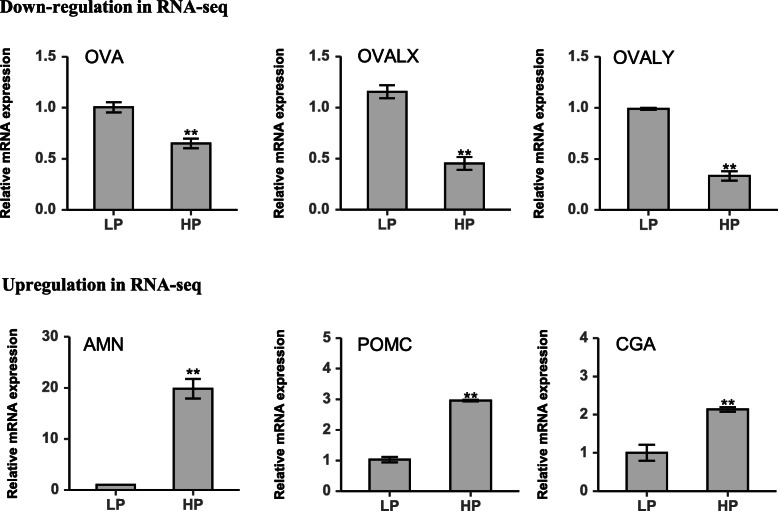
qRT-PCR validation of differentially expressed genes identified in transcriptome sequencing. The relative expression levels of genes were calculated according to the 2^−ΔΔCt^ method using β-actin as an internal reference RNA. **, *P* < 0.01. LP, low rate of egg production group; HP, high rate of egg production group

To validate the RNA-seq results, six DEGs were selected for qRT-PCR analysis. These included three up-regulated genes (AMN, POMC, and CGA) and three downregulated genes (OVA, OVALX, and OVALY). The results showed that the expression trends determined by the qRT-PCR were consistent with the RNA-Seq results (Fig. 6), indicating that the RNA-seq results were reliable.

## Discussion

To determine the differences in the ovary transcriptomes of the high and low-yielding layers, HP and LP groups were assessed. Their laying rates (%) were identified as 93.67 ± 7.09 and 68.00 ± 5.56, respectively, indicating that the animal model was appropriate. We noted that HP had a lower body weight than LP. It is well known that egg production is positively correlated with energy supply [13, 14]; thus, layers of the HP group may utilize more energy for egg production instead of body weight maintenance. In addition, previous studies have shown that egg production is negatively correlated with egg weight [15, 16], the eggshell thickness is positively correlated with strength [17], and similar results were observed in our study.

Egg production traits are determined by ovarian function and are regulated by the hypothalamic-pituitary-gonadal (HPG) axis [8, 18]. Thus, ovarian tissue was selected to perform the RNA-seq analysis. Significant differences were identified in the expression profiles of the ovarian tissues. According to KEGG pathway enrichment analysis, the neuroactive ligand-receptor interaction pathway comprised of multiple receptors that are associated with cell signaling [19, 20], was the most enriched. A previous study in fish found that the neuroactive ligand-receptor interaction pathway could affect steroid hormone synthesis in gonads through the HPG axis [21]. In our study, the ovarian steroidogenesis pathway was identified as one of the nine significantly enriched pathways, suggesting that the neuroactive ligand-receptor interaction pathway might affect egg production in chickens via a mechanism similar to that found in fish. Similar results were also previously reported for Jinghai yellow chickens [8]. In addition, Tao et al. [22] found that the same pathway was also involved in duck egg production. We identified eight DEGs that mapped to the neuroactive ligand-receptor interaction pathway (PRLR, FSHB, C3AR1, F2RL1, CGA, POMC, GALR1, and CCK) that may play an important role in the regulation of ovarian function and egg production. For the other seven significantly enriched pathways, the prolactin signaling pathway and GnRH signaling pathway have already been shown to affect avian egg production performance [23, 24]. The cAMP signaling and PI3K − Akt signaling pathways are reportedly involved in oocyte maturation and ovulation in mammals [25]. Further, the complement and coagulation cascades and inflammatory mediator regulation of the TRP channels that are associated with inflammatory responses [26], are required for ovulation in mammals [27, 28]. Thus, it is likely that these pathways may play similar roles in chicken ovaries, but further research is required to confirm this. Our results were not consistent with those of Mishra et al. [2] and Zhang et al. [8], who also conducted RNA-seq using chicken ovaries. Mishra et al. [2] identified four pathways that may play important roles in the regulation of egg production. Among them, PI3K − Akt signaling pathway alone was showed similarities with our results. Zhang et al. [8] reported five pathways, but only the neuroactive ligand-receptor interaction pathway was overlapped with our results. These conflicting results may be attributed to differences in the chicken breed used. In our study, Changshun green-shell layers were used, whereas in the Mishra et al. study [2], Chinese Luhua chickens were used, and in Zhang et al. [8], Jinghai yellow chickens were used.

The GO enrichment analysis showed that BP terms involved in regulation of peptidase and endopeptidase were most enriched. Peptidase is the term for any protein capable of catalyzing the hydrolysis of a protein substrate [29]. Endopeptidases cleave the interior region of the polypeptide chain. Thus, these results suggest that endopeptidases, but not amino or carboxypeptidases, may play a key role in the regulation of chicken ovary functions. The GO terms for the following biological processes showed significant enrichment: peptidyl-tyrosine modification, peptidyl-tyrosine phosphorylation, and positive regulation of peptidyl-tyrosine phosphorylation. The DEGs that mapped to these GO terms include PRLR, NRP1, IL15, BANK1, NTRK1, CCK, and HGF. The molecular function of these DEGs was transmembrane receptor protein tyrosine kinase activity. Protein tyrosine phosphorylation is the modification of post-translational proteins and plays a central role in many signaling pathways leading to cell growth and differentiation in animals [30]. Protein tyrosine phosphorylation is performed by a group of enzymes called protein tyrosine kinases, whereas receptor protein tyrosine kinases are a subclass of tyrosine kinases [31]. Using mouse ovaries, Hess et al. [32] showed that the receptor protein tyrosine kinase Ron was related to the regulation of ovary size and ovulation rates. Meanwhile, several studies have shown that the dysregulation of receptor protein tyrosine kinases is common in ovarian cancer [33–35], indicate the importance of receptor protein tyrosine kinases in the maintenance of ovarian function. Thus, the molecular function of transmembrane receptor protein tyrosine kinase activity as well as the DEGs involved in these GO terms, should be further studied in chicken ovary tissues.

Combing the results of the KEGG pathway and GO enrichment analyses, four DEGs were selected as candidate genes, i.e., PRLR, POMC, GALR1, and F2RL1. The first three have been shown to be involved in regulating egg production in chickens. PRLR (prolactin receptor) plays an important role in the PRL signal transduction cascade and is regarded as a genetic marker for reproductive traits in poultry [36]. POMC (pro-opiomelanocortin) is a member of the prohormone family and has an important function in regulating energy balance and reproduction [37]. A recent study reported that the POMC gene had potential effects on reproduction traits in chickens [38]. GALR1 (galanin type I receptor) is widely expressed in the chicken small intestine, kidney, ovary, pancreas, spleen and throughout the oviduct. A previous study indicated that GALR1 might be responsible for mediating chicken oviduct motility and follicle ovulation [39]. The association between F2RL1 and chicken egg production has not been reported yet. F2RL1 is involved in modulation of inflammatory responses and regulation of innate and adaptive immunity. Previous studies have indicated that inflammatory responses are the required processes for ovulation in mammals [27, 28], which implied that the expression level of F2RL1 may also affect the egg production in Changshun green-shell chickens.

In conclusion, we characterized and evaluated the ovarian transcriptome in low and high-yielding Changshun green-shell laying hens. A total of 142 differentially expressed genes were identified, which may serve as candidate genes for the genetic improvement of egg production. Moreover, enrichment analysis indicated that the neuroactive ligand-receptor interaction pathway and receptor protein tyrosine kinases may play crucial roles in the regulation of ovarian function and egg production.

## Supplementary Information

**Additional file 1:**

## Data Availability

The RNA-Seq datasets are available in the Sequence Read Archive of National Center for Biotechnology Information (https://submit.ncbi.nlm.nih.gov/subs/bioproject/; accession number: PRJNA685318).

## Abbreviations

DEGs: Differentially expressed genes
HP: High egg production group
LP: Low egg production group
PCA: Principal component analysis
KEGG: Kyoto Encyclopedia of Genes and Genomes
GO: Gene Ontology
PI3K − Akt: Phosphatidylinositol-3-kinase (PI3K) − protein kinase B (Akt)
cAMP: Cyclic adenosine monophosphate
GnRH: Gonadotropin-releasing hormone
TRP: Transient receptor potential
FDR: False discovery rate
BP: Biological processes
MF: Molecular functions
CC: Cellular components
AMN: Amnion associated transmembrane protein
POMC: Proopiomelanocortin
CGA: Glycoprotein hormones
OVA: Ovalbumin
OVALX: Ovalbumin-related protein X
OVALY: Ovalbumin-related protein Y
HPG: Hypothalamic-pituitary-gonadal
PRLR: Prolactin receptor
FSHB: Follicle-stimulating hormone subunit beta
C3AR1: complement C3a receptor 1
F2RL1: F2R like trypsin receptor 1
GALR1: Galanin receptor 1
CCK: Cholecystokinin
NRP1: Neuropilin 1
IL15: interleukin-15
BANK1: B-cell scaffold protein with ankyrin repeats 1
NTRK1: Neurotrophic tropomyosin kinase receptor 1
HGF: Hepatocyte growth factor
