# Supplementary material for: Transcriptome analysis of ovary tissues from low- and high-yielding Changshun green-shell laying hens
Source: BMC Genomics. 2021 May 14;22:349. doi: 10.1186/s12864-021-07688-x (PMC8122536; doi:10.1186/s12864-021-07688-x)
Supplement: Supplementary file 1 — Additional file 1: [file 12864_2021_7688_MOESM1_ESM.docx]

Table S1. The top 25 significantly enriched biological process GO terms

| ID | Description | Number of genes | p value | FDR | Gene |
| --- | --- | --- | --- | --- | --- |
| GO:0010951 | negative regulation of endopeptidase activity | 7 | 3.77E-06 | 1.96E-03 | SPINK7/ITIH5/RARRES1/SERPINB1/SPINK5/CRIM1/HGF |
| GO:0010466 | negative regulation of peptidase activity | 7 | 5.14E-06 | 1.96E-03 | SPINK7/ITIH5/RARRES1/SERPINB1/SPINK5/CRIM1/HGF |
| GO:0044060 | regulation of endocrine process | 4 | 5.26E-06 | 1.96E-03 | F2RL1/POMC/GALR1/GJA5 |
| GO:0052548 | regulation of endopeptidase activity | 8 | 1.34E-05 | 3.75E-03 | SPINK7/ITIH5/RARRES1/SERPINB1/CCK/SPINK5/CRIM1/HGF |
| GO:0052547 | regulation of peptidase activity | 8 | 2.09E-05 | 4.07E-03 | SPINK7/ITIH5/RARRES1/SERPINB1/CCK/SPINK5/CRIM1/HGF |
| GO:0048485 | sympathetic nervous system development | 3 | 2.19E-05 | 4.07E-03 | PLXNA4/NRP1/NTRK1 |
| GO:0032098 | regulation of appetite | 3 | 2.90E-05 | 4.63E-03 | POMC/CCK/CARTPT |
| GO:0045861 | negative regulation of proteolysis | 7 | 3.86E-05 | 4.96E-03 | SPINK7/ITIH5/RARRES1/SERPINB1/SPINK5/CRIM1/HGF |
| GO:0018108 | peptidyl-tyrosine phosphorylation | 7 | 4.22E-05 | 4.96E-03 | PRLR/NRP1/IL15/BANK1/NTRK1/CCK/HGF |
| GO:0018212 | peptidyl-tyrosine modification | 7 | 4.44E-05 | 4.96E-03 | PRLR/NRP1/IL15/BANK1/NTRK1/CCK/HGF |
| GO:0050886 | endocrine process | 4 | 6.92E-05 | 6.96E-03 | F2RL1/POMC/GALR1/GJA5 |
| GO:1902105 | regulation of leukocyte differentiation | 6 | 7.48E-05 | 6.96E-03 | IL15/IHH/FSHB/MYB/SPINK5/CARTPT |
| GO:1903532 | positive regulation of secretion by cell | 7 | 8.15E-05 | 7.00E-03 | ORM1/FGG/MYB/F2RL1/GALR1/CCK/CARTPT |
| GO:0048771 | tissue remodeling | 5 | 1.04E-04 | 8.14E-03 | IL15/IHH/FSHB/CARTPT/GJA5 |
| GO:0051047 | positive regulation of secretion | 7 | 1.18E-04 | 8.14E-03 | ORM1/FGG/MYB/F2RL1/GALR1/CCK/CARTPT |
| GO:0001818 | negative regulation of cytokine production | 6 | 1.19E-04 | 8.14E-03 | ORM1/BANK1/F2RL1/POMC/SERPINB1/HGF |
| GO:0030212 | hyaluronan metabolic process | 3 | 1.24E-04 | 8.14E-03 | ITIH5/IL15/HGF |
| GO:0050731 | positive regulation of peptidyl-tyrosine phosphorylation | 5 | 1.44E-04 | 8.95E-03 | NRP1/IL15/BANK1/CCK/HGF |
| GO:0140353 | lipid export from cell | 3 | 1.69E-04 | 9.92E-03 | MYB/POMC/GALR1 |
| GO:0051346 | negative regulation of hydrolase activity | 7 | 1.92E-04 | 1.07E-02 | SPINK7/ITIH5/RARRES1/SERPINB1/SPINK5/CRIM1/HGF |
| GO:1903510 | mucopolysaccharide metabolic process | 4 | 2.11E-04 | 1.08E-02 | EGFLAM/ITIH5/IL15/HGF |
| GO:0050708 | regulation of protein secretion | 7 | 2.16E-04 | 1.08E-02 | ORM1/FGG/BANK1/F2RL1/SERPINB1/CARTPT/GJA5 |
| GO:0048483 | autonomic nervous system development | 3 | 2.23E-04 | 1.08E-02 | PLXNA4/NRP1/NTRK1 |
| GO:2000849 | regulation of glucocorticoid secretion | 2 | 3.00E-04 | 1.20E-02 | POMC/GALR1 |
| GO:0002791 | regulation of peptide secretion | 7 | 3.07E-04 | 1.20E-02 | ORM1/FGG/BANK1/F2RL1/SERPINB1/CARTPT/GJA5 |

Table S2. The significantly enriched cellular components GO terms

| ID | Description | Number of genes | p value | FDR | Gene |
| --- | --- | --- | --- | --- | --- |
| GO:0062023 | collagen-containing extracellular matrix | 7 | 6.94E-05 | 3.10E-03 | EGFLAM/ORM1/FREM1/ITIH5/FGG/SERPINB1/C1QA |
| GO:0034774 | secretory granule lumen | 6 | 1.55E-04 | 3.10E-03 | ORM1/FGG/LYZ/POMC/SERPINB1/HGF |
| GO:0060205 | cytoplasmic vesicle lumen | 6 | 1.65E-04 | 3.10E-03 | ORM1/FGG/LYZ/POMC/SERPINB1/HGF |
| GO:0031983 | vesicle lumen | 6 | 1.71E-04 | 3.10E-03 | ORM1/FGG/LYZ/POMC/SERPINB1/HGF |
| GO:0005581 | collagen trimer | 3 | 1.40E-03 | 1.45E-02 | C1QTNF8/C1QA/C1QL2 |
| GO:0031091 | platelet alpha granule | 3 | 1.59E-03 | 1.45E-02 | ORM1/FGG/HGF |
| GO:0042581 | specific granule | 3 | 7.78E-03 | 5.67E-02 | ORM1/LYZ/C3AR1 |

Table S3. The significantly enriched molecular functions GO terms

| ID | Description | Number of genes | p value | FDR | Gene |
| --- | --- | --- | --- | --- | --- |
| GO:0004867 | serine-type endopeptidase inhibitor activity | 5 | 6.02E-06 | 2.67E-04 | SPINK7/ITIH5/SERPINB1/SPINK5/CRIM1 |
| GO:0004866 | endopeptidase inhibitor activity | 6 | 8.44E-06 | 2.67E-04 | SPINK7/ITIH5/RARRES1/SERPINB1/SPINK5/CRIM1 |
| GO:0030414 | peptidase inhibitor activity | 6 | 1.06E-05 | 2.67E-04 | SPINK7/ITIH5/RARRES1/SERPINB1/SPINK5/CRIM1 |
| GO:0061135 | endopeptidase regulator activity | 6 | 1.06E-05 | 2.67E-04 | SPINK7/ITIH5/RARRES1/SERPINB1/SPINK5/CRIM1 |
| GO:0005179 | hormone activity | 5 | 2.15E-05 | 4.34E-04 | FSHB/CGA/POMC/CCK/CARTPT |
| GO:0061134 | peptidase regulator activity | 6 | 3.01E-05 | 5.07E-04 | SPINK7/ITIH5/RARRES1/SERPINB1/SPINK5/CRIM1 |
| GO:0048018 | receptor ligand activity | 7 | 3.40E-04 | 4.57E-03 | IL15/FSHB/CGA/POMC/CCK/HGF/CARTPT |
| GO:0030546 | signaling receptor activator activity | 7 | 3.62E-04 | 4.57E-03 | IL15/FSHB/CGA/POMC/CCK/HGF/CARTPT |
| GO:0017154 | semaphorin receptor activity | 2 | 4.87E-04 | 5.47E-03 | PLXNA4/NRP1 |
| GO:0004857 | enzyme inhibitor activity | 6 | 5.64E-04 | 5.70E-03 | SPINK7/ITIH5/RARRES1/SERPINB1/SPINK5/CRIM1 |
| GO:0004714 | transmembrane receptor protein tyrosine kinase activity | 3 | 6.73E-04 | 6.18E-03 | NRP1/NTRK1/CRIM1 |
| GO:0019199 | transmembrane receptor protein kinase activity | 3 | 1.36E-03 | 1.15E-02 | NRP1/NTRK1/CRIM1 |
| GO:0005184 | neuropeptide hormone activity | 2 | 2.71E-03 | 2.11E-02 | CCK/CARTPT |
| GO:0004713 | protein tyrosine kinase activity | 3 | 6.07E-03 | 4.34E-02 | NRP1/NTRK1/CRIM1 |
| GO:0019838 | growth factor binding | 3 | 6.45E-03 | 4.34E-02 | NRP1/NTRK1/CRIM1 |
